# A novel insA2933 causes premature termination of translation and is accompanied by overexpression of truncated androgen receptor gene in a patient with complete androgen insensitivity syndrome

**DOI:** 10.1007/s13353-015-0288-3

**Published:** 2015-05-22

**Authors:** J. Turek-Plewa, J. B. Starzyk, W. H. Trzeciak

**Affiliations:** Department of Biochemistry and Molecular Biology, University of Medical Sciences, 6 Swiecickiego St., 60-781 Poznan, Poland; Department of Pediatric and Adolescent Endocrinology, Polish-American Children’s Hospital, Faculty of Medicine, Cracow, Poland

**Keywords:** CAIS, insA2933, (Y857X), Truncated AR, *AR* overexpression

## Abstract

A patient with a female phenotype, 46,XY karyotype, and a diagnosis of complete androgen insensitivity syndrome (CAIS) was examined. Her mother and three 46,XX sisters were also included in the study. Sequence analysis of the androgen receptor gene (*AR*) revealed a novel A2933 insertion that alters the Tyr codon to a termination codon (Y857X), resulting in a truncated form of the receptor. Computer simulation revealed major conformational changes in the hydrophobic pocket that accommodates the hormone. An insA2933 results in a truncated receptor incapable of binding the ligand and is responsible for the clinical symptoms of CAIS in the patient. The levels of the *AR* transcript in peripheral blood leukocytes were higher in the patient than in her heterozygous mother and her heterozygous sister, as well as in the two healthy sisters. It is hypothesized that elevated levels of the *AR* transcript in the patient might be caused by the inability of the truncated receptor to react with IFI-16, which functions in complex with AR to inhibit the expression of the *AR* gene.

## Introduction

Androgens such as testosterone (T) and dihydrotestosterone (DHT) affect the development and differentiation of the male external genitalia during embryonic development and are responsible for virilization of the pubertal male (Wilson et al. 1981). These effects are mediated by an androgen receptor (AR), which binds T and DHT with high affinity. Androgen receptor regulates transcription by interaction with specific androgen response elements (AREs) localized in the regulatory regions of target genes (Lee and Chang 2003). The androgen receptor gene (*AR*) is located on the X chromosome (Xq11-13) and contains eight exons (Quigley et al. 1995). Its protein product, AR, comprises an N-terminal part that is responsible for the antigenic properties of the receptor and contains the AF-1 sequence, which is responsible for activation of gene transcription. The DNA-binding domain (DBD), located in the center, contains two zinc-finger motifs and is responsible for binding to the AREs. The C-terminal part is comprised of the ligand-binding domain (LBD) involved in hormone binding, the AF-2 domain, the second sequence responsible for activation of target gene transcription, and the region responsible for dimerization of the receptor. From the crystalline structure of other members of the steroid receptor superfamily, it can be inferred that the LBD, encoded by exons 4 through 8, consists of 12 α-helices and two β-sheets, and includes a hydrophobic pocket that is located in the center of the molecule (Griffin et al. 2001; Wurtz et al. 1996).

Genetic defects that alter the function of AR can cause a wide range of abnormalities in male sexual development, and the effects of these defects are collectively referred to as androgen insensitivity syndrome (AIS).

These defects are transmitted in an X-linked recessive manner and have a prevalence of about 1 in 65,000 individuals. Three main phenotypic forms of AIS have been identified: a complete form (CAIS), a partial form (PAIS), and mild form (MAIS). All patients with CAIS have a 46,XY karyotype and possess a female phenotype with nearly normal female external genitalia and breast development. Instead of female internal genitalia, they have testes located in the abdomen or inguinal canals. Their pubic hair is scarce and axillary hair is absent. Testosterone levels are within the normal male range and estradiol is within the normal female range, while serum gonadotropin levels are normal or slightly elevated. The partial form (PAIS) is characterized by a wide spectrum of phenotypes, ranging from an incomplete female phenotype through various degrees of feminization of male external genitalia. In the mild form (MAIS), the phenotype is usually male and the symptoms of feminization can be very weak (Quigley et al. 1995; Sinnecker et al. 1997).

To date, more than 1,000 *AR* mutations have been described (Gottlieb et al. 2012). Most of these mutations are point mutations localized in the region encoding the LBD, particularly within helices V and IX. However, only about 30 mutations causing premature termination of translation due to generation of a termination codon and resulting in a truncated form of the receptor have been described (Gottlieb et al. 2012).

It has been demonstrated that androgens stimulate expression of a number of interferon-activated (*IFI*) genes belonging to the p200 family. Their protein products are known to inhibit cell cycle progression, modulate apoptosis, and act as negative regulators of gene expression, including the *AR* gene (Xin et al. 2003, 2004). In LNCaP prostate cancer cells transfected with a plasmid expressing *AR*, it has been reported that the *IFI-16* protein product binds within the DBD of AR and down-regulates *AR* and AR target gene expression (Alimirah et al. 2006). Up-regulation of *IFI-16* expression by AR could, thus, provide an important element of the feedback loop that negatively regulates transcription of the *AR*.

The present investigation was designed to identify molecular defects in *AR* in a patient diagnosed with CAIS and to estimate the level of *AR* expression in peripheral blood lymphocytes in this patient. An attempt was also made to explain why the expression of *AR* was increased in this patient.

## Case report

**Fig. 1:**
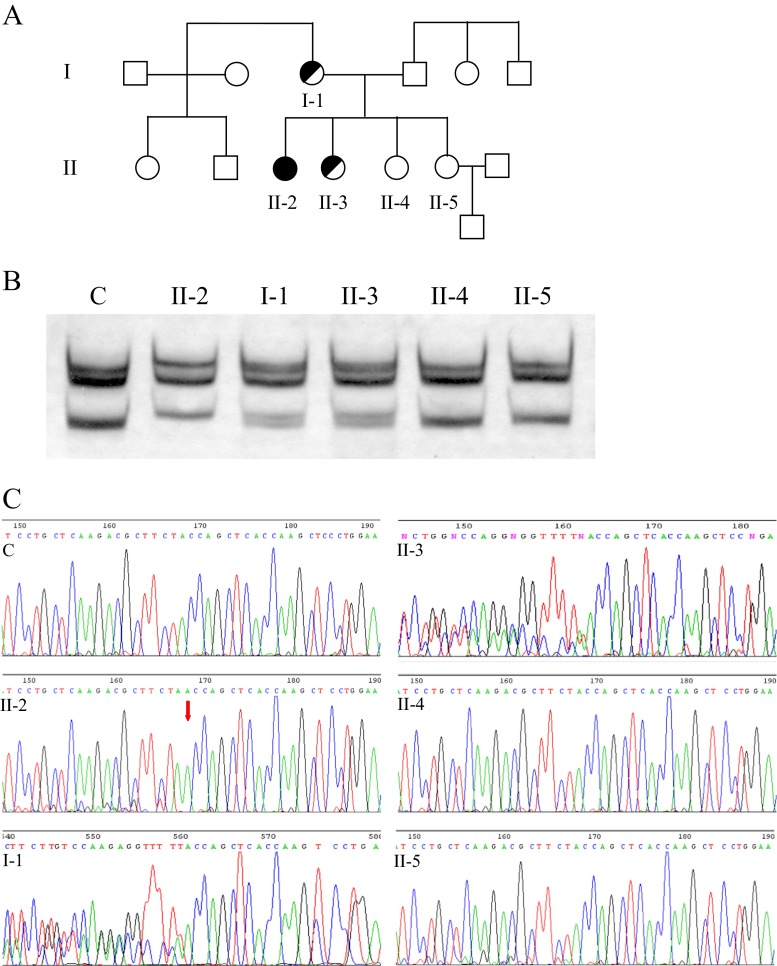
Pedigree of the family (**a**), multitemperature single-strand conformational polymorphism (MSSCP) analysis (**b**), and sequence analysis of exon F (**c**) of the *AR* gene. *I-1*, mother; *II-2*, patient; *II-3*, *II-4*, and *II-5*, healthy sisters; *C*, healthy individual (control). The position of insA2933 is indicated by an *arrow*

A 2-year-old girl (Fig. 1a, II-2) was admitted to the Department of Pediatric and Adolescent Endocrinology, Polish-American Children’s Hospital, Jagiellonian University, Cracow, Poland to correct a bilateral inguinal hernia. The child had unambiguous female external genitalia. The absence of female internal genitalia, combined with male gonads found bilaterally in the inguinal canals and a 46,XY karyotype, were consistent with the diagnosis of CAIS. Histopathological evaluation of the material removed revealed male gonad texture with lymphocyte infiltration. There was no history of sexual development disturbances in the family members. The patient’s mother (I-1) and three healthy sisters (II-3, II-4, II-5), referred to the hospital for genetic counseling, were also examined (Fig. 1). The University Ethical Committee approved the study and written consent from the patient’s mother was obtained.

## Materials and methods

Genomic DNA was extracted from peripheral blood leukocytes of the patient, her mother, and her three sisters. Exons 2 through 8 of *AR* were amplified by polymerase chain reaction (PCR) using Taq Polymerase (Fermentas, Lithuania) and previously described primers (Turek-Plewa et al. 2006). The PCR products were purified on DNA Gel-Out columns (A&A Biotechnology, Poland) and were screened using multitemperature single-strand conformational polymorphism (MSSCP) analysis to detect sequence-dependent differences in the electrophoretic mobility. A CEQ 8000 DNA analyzer (Beckman Coulter, USA) was used for direct DNA sequencing. In order to demonstrate conformational changes within the region of AR where the mutation was located, a structural analysis of the LBD was conducted with the use of Swiss PDB Viewer 3.7 software.

Total RNA was isolated from peripheral blood leukocytes (Chomczynski and Sacchi 1987) using TRItidy reagent (Applichem, Germany), and the RNA concentration was measured by spectrophotometry (Eppendorf, Germany). Poly A^+^ RNA was transcribed with the use of Moloney murine leukemia virus (MMLV) reverse transcriptase and random hexamers (Novazym, Poznan, Poland), according to the manufacturer’s instructions. AR and glyceraldehyde-3-phosphate dehydrogenase (GAPDH) cDNAs were amplified (RT-qPCR [reverse transcription quantitative PCR]) with the use of specific primers (Table 1).

**Table 1:** Primers used for the amplification of androgen receptor (AR) and glyceraldehyde-3-phosphate dehydrogenase (GAPDH) cDNA

cDNA	Forward primer	Reverse primer	Annealing (°C)	Product (bp)
AR	5′-acgacaacaaccagcccgac-3′	5′-gtgtaagttgcggaagccag-3′	60	122
GAPDH	5′-ttcgtcatgggtgtgaacc-3′	5′-gatgatgttctggagagccc-3′	60	231

Statistical analysis of the results was conducted with the aid of GraphPad InStat v.3.05 software and Microsoft Excel 2007. The results are presented as the mean (±SD [standard deviation]) of two repeats from three or more separate experiments and were subjected to one-way analysis of variance (ANOVA). All results were also tested with the post-hoc Student–Newman–Keuls test. The significance of the differences were tested at the level of *p* < 0.05, *p* < 0.01, or *p* < 0.001.

## Results and discussion

Diagnosis of the 2-year-old girl undergoing a procedure to correct inguinal hernia was based on the following clinical symptoms: unambiguous female external genitalia, the absence of internal female genitalia, male gonads found bilaterally in the inguinal canals, and 46,XY karyotype. Post-operative histological examination of the gonads was consistent with the diagnosis of CAIS, as no signs of Wolffian derivatives and male gonad texture were found.

Genetic investigation of the patient, her mother, and her three healthy sisters (Fig. 1a) involved screening for mutations in *AR* by MSSCP, followed by sequencing of the mutated fragment and structural analysis of the protein product of the gene in silico.

MSSCP analysis revealed an incorrect banding pattern of amplified exon 7 in the patient, as well as in her mother and in one of her 46,XX sisters, suggesting a possible mutation (Fig. 1b). Direct sequencing of these fragments (Fig. 1c) revealed an insA2993 mutation, which alters the Tyr857 codon UAC to form a UAA termination codon. This mutation leads to the truncation of the receptor by 62 amino acids at the C-terminal end, resulting in an 856-amino-acid protein devoid of a major part of the LBD. This mutation might result in an inability of ligand to bind to AR and may, therefore, be responsible for the clinical symptoms of CAIS found in the patient. The patient’s mother and one of the sisters (both carrying the 46,XX karyotype) are asymptomatic carriers of the same mutation. To date, only about 30 nonsense mutations in *AR* have been described (Gottlieb et al. 2012). In all cases, the truncation of AR was responsible for the symptoms of the disease. One of the previously described mutations located in the same position, a C2933G transversion, led to a frameshift and a premature termination of translation (Hiort et al. 1998). However, to date, no functional assay of this truncated receptor has been reported.

**Fig. 2:**
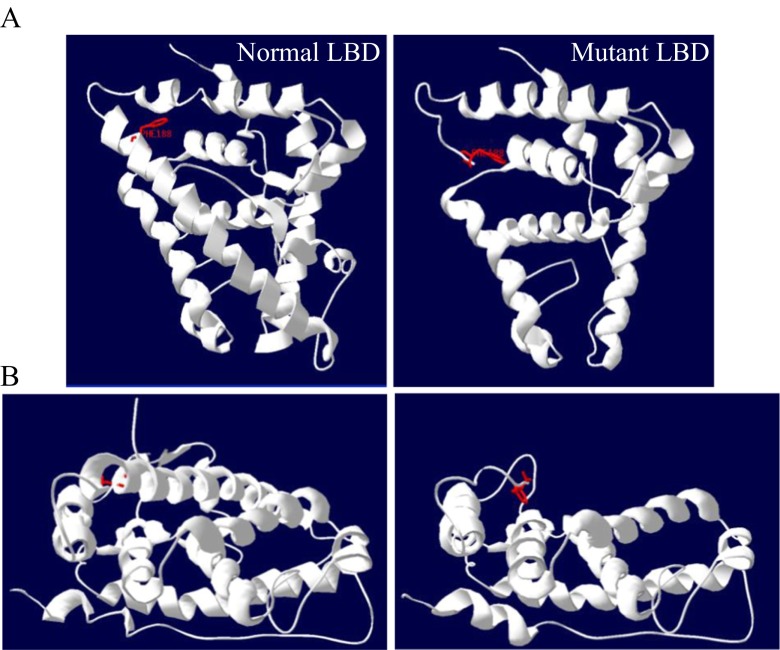
Structural analysis of the normal and mutated (Y857X) ligand-binding domain (LBD) of androgen receptor (AR) in the patient (Swiss PDB Viewer 3.7 software): **a** side view, **b** top view

Structural analysis with the use of Swiss PDB Viewer 3.7 software (Fig. 2) demonstrated that Tyr857 is located within the α-helix participating in the ligand binding. The truncated protein lacks a major part of the LBD which is responsible for the formation of the hydrophobic pocket and which, in turn, accommodates the ligand. The mutation also leads to abolishment of the β-pleated sheet that is normally located between helices VIII and IX. Profound conformational changes might severely impair or even abolish hormone binding by AR and may lead to the clinical symptoms of CAIS in the patient.

**Fig. 3:**
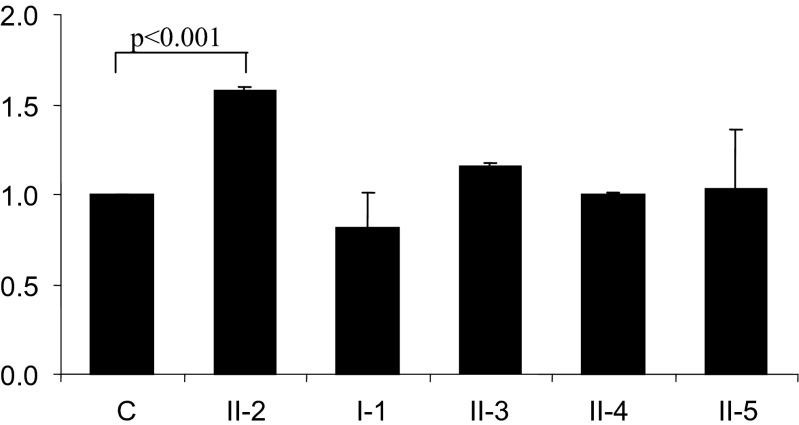
Relative levels of the *AR* transcript (A) in peripheral blood leukocytes. (A) mRNA was isolated from peripheral blood leukocytes, reverse transcribed, and the AR cDNA was amplified with the use of specific primers (RT-qPCR [reverse transcription quantitative PCR]), as detailed in the Materials and methods section. The results of individual measurements were normalized to glyceraldehyde-3-phosphate dehydrogenase (GAPDH) and the expression level in the normal individual was considered as 1.0. The results are represented as the mean of three estimations from three separate experiments. *C*, healthy individual (control); *II-2*, patient; *I-1*, mother; *II-3*, *II-4*, and *II-5*, healthy sisters

In order to investigate the influence of the insA2933 mutation on the expression of *AR*, the level of the illegitimate transcripts in peripheral blood leukocytes was estimated by real-time PCR in the patient, her mother, her three sisters, and in a healthy individual of the same sex. No difference in the *AR* transcript level between the patient’s mother and three sisters (including an asymptomatic carrier) was found, while in the patient, the *AR* transcript level was significantly higher (*p* < 0.001) than that seen in her mother and three sisters (Fig. 3a).

It has been reported that androgens acting via AR stimulate expression of *IFI-16*. The *IFI-16* protein product then interacts with the AR/ligand complex, which, in turn, inhibits expression of *AR* (Alimirah et al. 2006).

We hypothesize that the reason for higher *AR* expression in the 2-year-old girl with CAIS (II-2) than in her heterozygous mother (I-1), her heterozygous sister (II-3), and her two healthy sisters (II-4 and II-5) is that the truncated receptor, devoid of a large part of the LBD, is probably incapable of androgen binding and, hence, would be unable to stimulate *IFI-16* expression, because this requires a ligand–receptor complex. Therefore, the protein product of the *IFI-16* gene will not be formed and the IFI-16–androgen complex could not be formed and inhibit the expression of *AR*.

This hypothesis, however, should be substantiated by further research using a group of CAIS patients with mutations leading to truncation of the LBD of AR.
